# FaTEMa: A Framework for Multi-Layer Fault Tolerance in IoT Systems

**DOI:** 10.3390/s21217181

**Published:** 2021-10-29

**Authors:** Mário Melo, Gibeon Aquino

**Affiliations:** 1 Academic Department, Federal Institute of Rio Grande do Norte, Lajes 59535-000, Brazil; 2 Department of Informatics and Applied Mathematics, Federal University of Rio Grande do Norte, Natal 59078-970, Brazil; gibeon@dimap.ufrn.br

**Keywords:** IoT, dependability, fault tolerance, error detection, error recovery, reliability, availability

## Abstract

Fault tolerance in IoT systems is challenging to overcome due to its complexity, dynamicity, and heterogeneity. IoT systems are typically designed and constructed in layers. Every layer has its requirements and fault tolerance strategies. However, errors in one layer can propagate and cause effects on others. Thus, it is impractical to consider a centralized fault tolerance approach for an entire system. Consequently, it is vital to consider multiple layers in order to enable collaboration and information exchange when addressing fault tolerance. The purpose of this study is to propose a multi-layer fault tolerance approach, granting interconnection among IoT system layers, allowing information exchange and collaboration in order to attain the property of dependability. Therefore, we define an event-driven framework called FaTEMa (Fault Tolerance Event Manager) that creates a dedicated fault-related communication channel in order to propagate events across the levels of the system. The implemented framework assist with error detection and continued service. Additionally, it offers extension points to support heterogeneous communication protocols and evolve new capabilities. Our empirical results show that introducing FaTEMa provided improvements to the error detection and error resolution time, consequently improving system availability. In addition, the use of Fatema provided a reliability improvement and a reduction in the number of failures produced.

## 1. Introduction

The Internet of Things has emerged as a framework of technologies and paradigms for the future of the Internet [1]. The term Internet of Things (IoT) has attracted attention by projecting the vision of a global network of physical objects, enabling connectivity anytime, anywhere, for anything and anyone [2,3,4]. The IoT has made everyday life easier through technology [5]. However, it comes with a price: failures in those systems can cause fatal accidents, undermining their trustworthiness in the public eye [6]. For this reason, the dependability of IoT systems has become crucial in many contexts, particularly in critical domains [7,8]. Although much progress has been made in such systems, hardware, approaches, and networks, several challenges still need to be appropriately addressed [9,10,11].

The means to address dependability are fault prevention, fault tolerance, fault removal, and fault forecasting [12,13]. Fault tolerance (FT) plays an essential role in maintaining a system’s regular operation, even in the presence of failures, leading to less faults, errors, and failures, improving the dependability [14]. However, applying fault tolerance techniques is a complex task, posing these challenges: IoT systems are often loaded with multiple heterogeneous "things" to support multi-purpose applications [15,16,17]; they often have particular requirements regarding fault-tolerance schemes, including specific fault characteristics [18,19]; complexity due to large-scale and ubiquitous applications [6,10,20]; and they are in constantly evolving [21,22,23].

Current implementations of FT mechanisms in IoT are focused on specific problems: (1) they are designed for a specific architecture and application [24,25]; (2) they do not scale beyond small decentralized [26,27] and centralized [24] solutions; and (3) they provide solutions to specific faults, such as link failures [28] and device failures [29], and they often focus on one layer [30,31]. Moreover, different layers in IoT systems operate cooperatively, wherefore data and information generated in one layer are usually distributed across different layers for further transmission or processing [32,33]. Therefore, errors in one layer can propagate, causing faults in other layers and are likely to cause the system to fail as a whole. In addition, the decision applied to tolerate to a given fault can impact the functioning of other layers or services. Nevertheless, due to the present challenges in IoT, it is hard to adopt a unified and effective fault tolerance mechanism to contemplate all system layers [18,34,35]. Besides, it is unlikely, even impossible, to assure that every layer will handle all errors and faults. Therefore, it is vital to consider multiple layers from a holistic viewpoint, addressing fault tolerance information exchange to improve the system dependability.

This paper proposes a multi-layer approach to assist the information sharing between fault tolerance mechanisms in IoT systems. It focuses particularly on error detection and service continuation steps through a transversal and interoperable communication channel. The term "multi-layer fault tolerance" refers to the idea that it is better suited to increasing system dependability with respect to fault tolerance by reasoning about system layers as a whole, rather than by completely abstracting the operation of individual layers and trying to improve the efficiency of each of them in isolation [31,36,37,38]. Thus, the objective is to minimize the effort expended by hardware, software, and the network, and the complexity, through information and adaptation, along with collaboration between the layers, compared to cases in which the layers are concerned with tolerating faults without knowing their own context. Consequently, this should reduce the occurrence and propagation of errors, improving system reliability and availability [39,40]. Distributing fault tolerance throughout the system stack will allow designers to implement each aspect of fault management most efficiently. Therefore, our hypothesis is that the adoption of multi-layer fault tolerance in IoT systems can produce more dependable systems by distributing the responsibilities of tolerating faults across multiple layers.

In order to validate the aforementioned hypothesis, we implemented a framework called FaTEMa (Fault Tolerance Event Manager). This framework uses an event-driven architecture providing extension points that enable ways to meet each level’s inherent needs and enable heterogeneous intercommunication. In addition, it acts as a dedicated channel for the transversal communication of fault tolerance information and its implications. We also defined a standardized and adaptive communication format to support the heterogeneity and evolution of technologies and protocols. The responsibility for fault tolerance lies in each layer and system, considering its requirements and constraints. Thus, FaTEMa facilitates information sharing about faults to be propagated across system layers in a simplified, transparent, and unified way.

To evaluate the FaTEMa’s feasibility and effectiveness regarding improving IoT system dependability, we designed an experiment to empirically compare a system implementation with well-known fault tolerance techniques using FaTEMa and one not using it. Our experiment simulated a smart home system. We injected two faults to measure and analyze each scenario regarding error detection and resolution, availability, and reliability. Our results indicate that FaTEMA has better efficiency in error detection and recovery when compared to popular techniques, improving the system’s availability and reliability, and consequently, the overall system dependability.

The contributions of this paper are summarized as follows:

- We introduce a novel mechanism which supports multi-level fault tolerance, which supports information exchange among different fault tolerance strategies, especially for error detection, error recovery, and continued service.
- We implemented a novel framework called FaTEMa. This framework uses an event-driven architecture providing extension points to ensure ways to meet the inherent needs of each layer and enable heterogeneous intercommunication.
- We performed an empirical study based on a smart home system in order to evaluate the adoption of our approach.

The remainder of the article is organized as follows: In Section 2, we present fault tolerance in IoT challenges and revise related works, pointing out their weakness and strengths. Section 3 discusses the design choices and our approach. In Section 4, we present the novel multi-level fault tolerance approach and the FaTEMa framework in detail. In Section 5, we empirically evaluate the proposed approach, comparing two scenarios in a smart home system. We also discuss the findings. Finally, we summarize the paper’s findings and present research gaps in Section 6.

## 2. Challenges and Related Work

In this section, we first outline some of the main challenges that exist for designing and developing fault tolerance approaches in IoT systems, and then we present the findings of our study on fault tolerance approaches to motivate the need for a multi-layer fault tolerance approach.

### 2.1. Main Challenges

Fault tolerance in IoT systems is a challenge. From a literature review, we list the main challenges faced when building approaches to perform fault tolerance in such systems.

#### 2.1.1. CH1—Heterogeneity

Heterogeneity is a common characteristic of IoT systems, as they usually involve different technologies, devices, forms of communication, and protocols [41,42]. IoT systems typically organize their infrastructures into layers that interact with each other. Each layer has specific requirements and constraints, and fault tolerance mechanisms, focused on attaining their requirements [18,30]. This provides a unified fault tolerance mechanism that integrates system layers, and their heterogeneity becomes a challenge to be overcome. Therefore, fault tolerance strategies must have mechanisms that enable integration and collaboration to provide more efficient and effective fault tolerance solutions [33,43,44].

#### 2.1.2. CH2—Adaptability and Evolution

New devices, technologies, and systems can be introduced, removed, paused, resumed, or retired in most IoT deployments. Changes will occur over time, generating a need for fault tolerance mechanisms that support this evolution and flexibility [6]. In addition, many systems need real-time responses, given that they work in critical contexts, or even are responsible for attending requirements on which people’s lives depend, and cannot be stopped whenever a change happens. Therefore, tolerance mechanisms must have ways to provide adaptation to new characteristics and requirements at runtime [23,45].

#### 2.1.3. CH3—Distributed Decision Mechanism

Applying a fault tolerance approach to one layer could cause impacts on other layers or even in different systems. The use of a centralized decision-making approach in IoT systems is impractical due to the complexity, heterogeneity, and large scale [39,46]. Thus, fault tolerance approaches must have knowledge and information about decisions made in other layers, allowing the decision-making process to occur in a decentralized manner, attaining the dependability requirements required at each layer [18,39,47]. Detecting faults and resuming system operation after their treatment without a complete view of the system is a challenge, which is why fault tolerance mechanisms must support the delegation of the decision-making process to closer entities with more knowledge about the error [47,48].

#### 2.1.4. CH4—Information Redundancy

In IoT systems, hardware redundancy is impractical in large-scale applications with thousands or even millions of devices. Furthermore, the number of devices is increasing, and the number of interactions is becoming more significant, leading to failures. Therefore, the inclusion of hardware redundancy can lead to increased system complexity and cost [40,45]. Redundancy is an essential part of the fault tolerance process and can mask a fault or assist in the detection stage [49]. Therefore, redundant information propagation between system entities must be made possible without any increases in the costs and complexity of the solutions, improving fault tolerance strategies. Thus, it is necessary to enable the redundancy of information without the need to include new devices or including the smallest amount possible [50,51]. Although the redundancy on failure information does not guarantee that abstractions suffer from errors and defects, it motivates the diversity of threat treatment [52]. In this way, the different layers of the system will be able to use their measures to avoid the occurrence of failures [53].

### 2.2. Related Work

Different authors have suggested quite diverse approaches and techniques; however, the heterogeneity, complexity, and large scale make discovering and treating faults large challenges to overcome. The subject of fault tolerance in IoT systems has been a hot topic in recent years. Therefore, we realize an extensive analysis of the existing literature to identify research that addresses the main challenges to achieving fault tolerance in IoT systems. Thus, we list and summarize them in Table 1, which compares the challenges addressed by the related works and the approach proposed in this work.

Li et al. [18] proposed a layer-based framework for fault detection, location, and recovery for heterogeneous IoT systems in order to unify fault measures and maximize existing resources. For this purpose, they used fuzzy cognitive maps, allowing adaptive monitoring model of the observed points. Fault management was performed through a layered scheme in which the first layer includes detection and location steps. Several observation points were defined in this layer that monitored and analyzed the communication links using FCM-advanced to predict the risk of a failure. When one of the observer points identified that a failure had occurred, the second layer activated failure recovery mechanisms across the network. As a result, the identification of failures occurred in a distributed way, but the recovery occurred in the specific network. The authors evaluated the framework using a scenario containing three types of networks: wireless sensor networks, wired networks, and Wi-Fi networks. The evaluation was carried out by comparing the proposed scheme with a traditional destination reporting algorithm in which aspects related to fault location time, transmission time, and probability of false alarms were evaluated. The results presented by the authors demonstrate the suitability of the proposed fault management schema. It improves fault detection and reduces the probability of false alarms.

Fortino et al. [54] defined a framework to address the interoperability issue in the IoT domain by proposing a complete approach that aims to facilitate "voluntary interoperability" at any level of a system and across domains of any IoT application, ensuring simplified integration of heterogeneous IoT technologies. The INTER-IoT approach includes three solutions to provide voluntary interoperability. (1) INTER-LAYER: creates a layer-oriented approach providing interoperability and exploiting layer-specific functionalities. (2) INTER-FW: this component provides a global and open platform to manage the interoperability between IoT platforms acting over the INTER-LAYER. (3) INTER-METH: defines a methodology for the process of building interoperable IoT platforms.

The authors demonstrated how the framework works by describing three use cases. Use cases for container transport in a smart port, decentralized monitoring of assisted living, and integration between health monitoring platforms were presented. Despite describing the usage scenarios, the proposed framework lacks evaluation and implementation in simulated or real environments to prove its functioning and possible problems.

In Abreu et al. [55], the authors proposed an end-to-end resilient IoT architecture for smart cities. Their contribution was to define an architecture that considers the design, implementation, and protocols that support key dependability features in different architecture layers. The proposed architecture is composed of three layers: IoT infrastructure, IoT middleware, and IoT services. One of the key features is the possibility of having more than one instance per layer. Consequently, the services of a layer can be instantiated several times. In addition, for more ubiquity and flexibility of components, the middleware and service layers reside in the cloud. Furthermore, the architecture supports the virtualization and deployment of essential components to reduce the latency of critical applications. The main features included are: The heterogeneity manager, which enables intercommunication between physical devices and the cloud, translating data using heterogeneous protocols in the lower layer to a common language. The communication manager, which establishes how the information will be exchanged between services and applications and smart objects. This component works by providing route control, communication infrastructure, entity mobility, and specific requirements of the IoT context. A virtualized device manager, which provides device administration enabling the identification, discovery, and location of services. A resilience manager, which provides reliability for the IoT infrastructure. In addition, it offers protection and recovery mechanisms that work together with path control, topology, and mobility control mechanisms to be applied in case of failures. This component also orchestrates heterogeneous dependability techniques, enabling recovery of the IoT infrastructure. IoT services manage applications and services that are supported in smart cities. In addition, it makes it possible to analyze the data collected by sensors using big data.

The authors did not present a concrete implementation. They only described the functioning of the architecture through scenarios of failures occurring and the system recovering from them. However, the description is succinct, and it is not possible to prove the functioning of the architecture; and there is no clear evidence that its use improves the dependability of IoT systems.

Woo et al. [25] researched, proposed, and built an IoT system for personal healthcare devices (PHD) based on the oneM2M communication protocol. However, using PHD in oneM2M systems is necessary to perform the conversion between protocols. Thus, to guarantee the operation with the different IoT servers, they used the ISO/IEEE 11073 protocol supported by most PHDs, thereby requiring a translation mechanism. The proposed system is divided into an application dedicated node-application entity which collects and transmits data using the ISO/IEEE 11073 protocol. The collected data are transmitted to a centralizing entity (middle node-common service entity—(MN)) or a PHD (infrastructure node-common service entity) management server. The latter is responsible for centralizing the information and making it available using the oneM2M protocol. Translation also takes place in the communication between the PHD and the MN.

Concerned with faults that might occur in gateways, a fault-tolerant algorithm was proposed to increase the system’s reliability. The algorithm uses a hierarchical network that connects gateways from the same layer and immediately above the layer, forming an interconnected chain. Each gateway stores a copy of the gateway previously placed in the chain. The data from the last gateway in the chain are stored in the next higher layer gateway. This way, even if a failure occurs in two gateways simultaneously, it can be recovered. The proposed system and algorithm were evaluated through experiments with multiple hypothetical scenarios, revealing that it was possible to recover from failures.

Despite showing that the system could recover from a failure, the authors did not demonstrate a benefit of using the algorithm to recover the system by measuring the time it took to recover it. This information is crucial for assessing system availability. Another aspect the authors mention is the increased complexity of implementing this solution in an environment composed of several layers and devices. In addition, the fault tolerance mechanism is focused only on the recovery process. It is not clear which steps or processes allow for identifying failures and how the proposal improves this step.

Consequently, it is not clear that the upper layers can make decisions about fault tolerance, since they only have data from the layers immediately below. Furthermore, the fault tolerance mechanism does not consider devices and sensors as parts of the fault recovery process. The proposed solution, although promising, is focused only on the healthcare area and uses specific protocols, not hindering the inclusion of new technologies and protocols.

Belkacem et al. [56] proposed in their work an approach to improving the dependability of IoT systems, using fault tolerance and statistical data from several remotely distributed locations equipped with redundant communication technologies (such as RFID, NFC, and beacons) and sensors for monitoring context and environments. This approach aims to detect and correct failures that occur, especially in locations where local fault tolerance (LFT) is unreliable, or where there is no LFT system. A central server performs reliability and error correction.

To improve dependability, the authors explored collaboration between various remote locations, thereby profiling the behavior of each identifying node and sensor node. This profiling process makes it possible to select the most reliable nodes from a comparative study between data collected from different locations and nodes with similar conditions. Decision-making can occur in a distributed or centralized way. A central server (common to all remote locations) diagnoses failures based on profiling and collected data in the centralized approach. This approach ensures complete monitoring of network status to ensure a more accurate error diagnosis. Thus, to limit this dependence on the central server, fault detection is also performed in a distributed manner at each remote location. Therefore, if the local server does not have an LFT or has an inconclusive LFT, the decision-making is transferred to the central server, detecting a failure and correcting it. Failure detection occurs through a statistical analysis involving the detection of outliers and the correction of extreme studentized deviate (ESD) test data. The proposal presented by the authors was not evaluated in a simulation or assessed in a real system. The authors only compared their proposal with others existing in the state-of-the-art, considering aspects such as data analysis, data correction, and decision-making.

Guimaraes et al. [33] proposed a framework (IoTUS) that allows information sharing between layers while preserving layering benefits, such as modularity and portability. The framework defines a transversal and extensible service layer allowing information exchange regarding the system’s functioning (e.g., numbers of transmissions, receptions, and collisions in the data-link layer) and services (e.g., neighbor discovery and data aggregation). The framework can be used with existing communication protocols, as it works by intermediating communication and building new packages that will be exchanged between layers, including metadata information. It has a set of modules that act in various functions, such as discovery, routing, package assembly, and synchronization. It aims to improve the system’s power consumption. However, introducing extra communication processing causes an increase in CPU consumption. All adaptation and interoperability must be defined at design and compile time to define which protocols will be supported. The authors evaluated the framework without considering the latency introduced by the solution. The results showed that the IoTUS approach achieved better performance in energy consumption when compared to other state-of-the-art approaches.

In Su et al. [57], a framework which provides a decentralized fault tolerance mechanism was proposed. The developed mechanism aims to detect failures, recover from them, and dynamically reconfigure the system. This mechanism aims to provide a failover for the system components (services) meeting the fault tolerance requirements by adopting a decentralized mechanism that avoids a single point of failure and performance bottlenecks. The decentralized mechanism uses a service replication mechanism to support fault tolerance, in which each component is replicated in other devices, thereby creating a “redundancy level”. Each device monitors the other by forming a daisy monitoring chain through heartbeat checks. Consequently, when a device does not send its information through the heartbeat message, the device update process is activated, thereby removing the faulty device from the chain and delegating to the “redundant” devices the components necessary for the system to return to operating normally.

The authors evaluated the performance of the proposed mechanism through an experiment. This experiment considered ten devices mapped into four nodes. The first metric evaluated was message overhead during device failure. It took about 550 bytes of data to recover from a failed node. The second metric evaluated by the authors was average recovery time for nodes, which was approximately 2500 ms. The third metric combined the detection time with the recovery time and could not have a time greater than the heartbeat time, which was confirmed by the presented data. Despite demonstrating the feasibility of the mechanism, the authors did not raise or indicate concerns regarding interoperability and adaptation to new technologies.

Furthermore, in some cases, the recovery mechanism took more than 3500 ms to recover from a failure using ten devices. However, it cannot achieve efficient scalability in a real scenario with thousands or even millions of devices. The authors did not demonstrate complexity or overhead impact on the overall system functioning due to the introduction of the decentralized tolerance strategy.

## 3. Design Decisions

We approach, in this Section, the main challenges reported for building fault tolerance mechanisms in IoT systems (see Table 1). Hence, we will present the design decisions applied in the FaTEMa framework to overcome these challenges. For each decision, a general approach will be described and then we will discuss how it was implemented.

### 3.1. Multi-Layer Communication Channel

IoT systems usually have modular structures, divided into layers or levels of abstraction. These layers may each have a set of devices, systems, technologies, and fault tolerance approaches. Therefore, the dependability of IoT systems could be established by considering the sum of the dependability present in each layer. However, this simplistic assumption does not take into account collaboration between layers that typically produce information that is transmitted and processed by others [30,32,33]. Furthermore, without knowledge of the status or information about events linked to failures of other layers, it is impossible to adjust the functioning of a layer to maximize the optimal operation of the system, making the fault tolerance process difficult [31,38]. Another crucial factor to be considered is that each layer will be able to handle and mitigate all types of errors that can happen, either in the layer itself or in layers adjacent to it. This type of situation leads to the generation of the phenomenon of fault propagation. This type of behavior causes a chain reaction or domino effect, causing extensive damage and the breakdown of the entire system [11]. The first step to avoid cascading is error detection [58].

In our approach, we create a transversal communication channel across layers, allowing information exchange between system layers. Consequently, this channel enables collaboration among specific layer fault tolerance approaches to avoid error propagation, improving error detection and service continuation. Thus, each system layer must have at least one instance of FaTEMa to ensure this transversal communication. In addition, each layer will act on the error assertively and align with its requirements because, besides the information from other layers, it will use its contextual information and apply the best strategy (CH4).

### 3.2. Interoperable Communication

Due to the heterogeneity of devices and protocols present in IoT systems, there is no standardized communication method, hindering the collaboration process between the layers and their FT approaches [6,59]. Therefore, to enable this intercommunication and support the heterogeneity of devices, it is necessary to establish a unified communication model that meets system requirements and does not increase complexity or system overhead [21].

In our approach, we define a structured communication approach indicating a communication format regarding standardized fault tolerance events. This communication is premised on technology independence and includes the minimum control information (CH1). Therefore, it will be possible to transfer information between the different layers of an IoT system. In addition, FaTEMa will provide extension points allowing operation with different communication protocols. Thus, it will be possible to support the heterogeneity of protocols and possible evolutions or adaptations in the systems.

### 3.3. Event-Driven Architecture

IoT systems are intrinsically associated with environments. The environment is constantly changing, as are the systems themselves due to the inclusion of new technologies and protocols [47]. This dynamic and constant change directly impacts the dependability of systems, requiring mechanisms that support adaptability [55,60]. Thus, fault tolerance approaches must operate while considering this scenario of constant change and unpredictability. Therefore, considering the set of layers present in IoT systems, it is necessary to develop a solution that can provide mechanisms to support such changes while meeting the needs of dependability and fault tolerance [21]

In our approach, aiming to support adaptability and evolution, FaTEMa uses an event-driven architecture. All communication is asynchronous and application-independent. For this reason, FaTEMa should have a limited possible impact on the existing infrastructure. Another factor that favors this type of architecture is the adaptability to new technologies or evolution, since events are generic to support any data format (CH2).

### 3.4. Collaborative Error Detection and Recovery

IoT systems are an interrelated set of devices and entities that work collaboratively to obtain and process information. This relationship causes an interdependence between devices, services, layers, and systems resulting in complex interactions among such entities. Thus, the occurrence of a failure in a given component can propagate and lead to failure of other dependent components [61]. This dynamic behavior requires mechanisms to avoid the cascading and propagation of faults between entities, identifying, isolating, and recovering such errors [11,30]. Thus, the collaborative and distributed error detection and recovery process assist the fault tolerance strategies to manage and make better decisions for the system to return to its proper functioning.

In our approach, we provide means to support a multi-layer approach to propagate fault tolerance information across layers, enabling collaboration in the error detection and recovery steps. Our approach focuses on providing information redundancy across the layers, enabling decentralized decision-making according to the restrictions and requirements of each layer (CH4). Furthermore, fault tolerance information produced in other layers can be combined with contextual information, enabling the construction and design of more efficient and effective fault tolerance strategies to improve applications’ dependability.

## 4. FaTEMa Framework

Our approach consists of a multi-layer communication channel that allows the exchange of events related to fault tolerance aspects. This channel assists and allows collaboration of the different fault tolerance strategies without interfering in the system’s functioning. In order to instantiate the proposed approach, we developed the FaTEMa (Fault Tolerance Event Manager) framework. It consists of extensible components to handle external fault tolerance events and propagate them across layers of an IoT system. Moreover, it acts as a mediator, enabling communication between IoT system levels to assist fault tolerance mechanisms, aiming for failure avoidance. Thus, it facilitates information exchange between local or distributed techniques, providing interoperability to improve systems’ dependability.

The use of fault tolerance aims to prevent faults from leading to system failures. Fault tolerance has four constituent phases: error detection, damage confinement and assessment, error recovery, fault treatment, and continued system service [49]. FaTEMa focuses on assisting error detection, fault treatment techniques, and continued system service, especially the latter.

The success of any fault tolerance technique depends on the effectiveness of detecting errors. Thus, the initial stage of any technique is the error detection step. Therefore, the more errors detected, the better the system’s dependability will be. Once the errors are detected, the appropriate techniques can be applied to prevent the system from failing. For this reason, the use of FaTEMa allows the propagation of information regarding error detection, allowing different levels and techniques to cooperate in order to recover from those errors, which could eventually avoid a system failure.

Once an error is detected and recovered, it is necessary to return a system to its regular operation. Consequently, components could be resumed or restart, others can be reconfigured, and some can be terminated. Once an error is detected and recovered, it is necessary to return a system to its regular operation. Consequently, components could be resumed or restarted, others can be reconfigured, and some can be terminated. Hence, FaTEMa assists in this error recovery and service continuation process, allowing the mechanisms to make available and share information between the layers so that the most appropriate measures are taken, and the system resumes its regular operation.

However, FaTEMa, by itself, does not contain any fault tolerance mechanism. The FaTEMa framework’s purpose is to enable intercommunication between the various levels of abstraction, enabling efficient and effective error detection, besides the service continuation. Additionally, the proposed framework aims to be a technology, hardware, or operating system agnostic, facilitating the development of new solutions for fault tolerance.

### 4.1. Architecture

**Figure 1:**
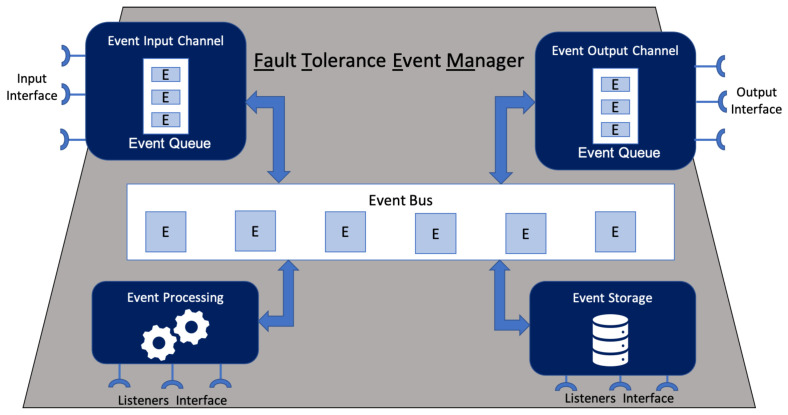
Fault Tolerance Event Manager (FaTEMA) Architecture.

Figure 1 depicts the FaTEMa architecture. It is composed of five components: event input channel, event processing, event storage, event output channel, and event bus. They act independently, with well-defined responsibilities. Moreover, the communication between them occurs through asynchronous messages posted and received at the event bus. This interaction between the components and the event bus occurs using the publish–subscribe messaging pattern [62]. Thus, the components subscribe on the bus to certain types of events. Once the events of interest are detected, the components are notified. Additionally, each component can send events to the event bus. However, there are two special cases: event input channel and event output channel. The former can only post external events received at the entrance of the framework in the bus. The latter is only allowed to receive events posted on the bus and make them available externally.

In order to ensure scalability and layer independence, the framework’s architecture was designed to not store states, working in a stateless way. Thus, if it is desirable to store or retrieve event information, it should be explicitly implemented, making use of the event storage component (detailed in Section 4.1.3). Furthermore, the events moving through FaTEMa are non-persistent, which means that the framework only receives, processes, and distributes them. However, if the communication protocol for sending and receiving events establishes persistence and replication strategies, this behavior will be incorporated into FaTEMa.

Additionally, each component has extension points to allow new behaviors to be introduced. The framework uses the whiteboard pattern [63], allowing the extensions to indicate the events of interest. Once the component receives an event, it is transferred to the corresponding extension point if it exists. If there is no one interested in the event, it is placed on the event bus to be made available to the next component of the architecture. This architectural design was inserted due to the constant change and evolution present in IoT systems. Thus, FaTEMa allows the inclusion of new technologies and protocols. Furthermore, it is possible to evolve the framework in order to support new types of events since its structure has an extensible format. Moreover, there is no coupling and dependency between FaTEMa’s aiming to prevent the interference of one instance of the framework with another. A FaTEMa only has to know the addresses of the underlying FaTEMa layers. Therefore, each layer may have a different framework instance implementation and functionalities to meet their current needs.

#### 4.1.1. Event Input Channel

This acts as an event broker, providing an external communication interface to receive events. In order to support heterogeneous communication, the component provides an extensible interface allowing the implementation of different communication protocols. However, due to the heterogeneity of technologies in IoT, this component defines a standard event structure, ensuring interoperability between protocols. In particular, each event must contain the following fields:

- Identifier (64 bits): Identifies uniquely a FaTEMa message in an IoT system.
- Direction (2 bits):Defines the direction of event propagation. It can assume three values: 0—both ways, 1—upward and, 2—downward.
- Type (2 bits): By default, there are two types: control and notification. However, this field supports expansion for other purposes.
- Subtype (4 bits): The fault tolerance strategies use this field in order to exchange information. A fault tolerance approach must subscribe to receive a specific event subtype.
- Source (128 bits): Indicates the event sender address. The field represents the entity’s unique identification which is sending the event to the framework. Each system is responsible for generating its entity identification numbers.
- Probable Destination (128 bits): Indicates a probable destination of an event. However, this field is optional. To apply in an IoT system should be assured that every entity has a unique address. If the destination address does not exist or is unavailable, the event will go through the entire framework flow and be made available to interested parties.
- Payload (0–512 bytes): It contains data related to fault tolerance. This field allows engines to share information about the various events related to failures. For example, if a sensor is failing, the information about this error occurrence could be made available, and it could be informed that there is a spare sensor that can be used in its place. Thus, this event will be made available so that the other layers could act by adjusting their functioning to this new reality.

#### 4.1.2. Event Processing

This component addresses the event processing capabilities. The events received are forwarded to the extensions to be processed and sent to the event bus. The event processing can be the collection of information, registration of devices, and exchange of information regarding the status of FaTEMa, among others. However, it is essential that this processing is not dependent on other systems, given that FaTEMa acts in a distributed manner and independently of other systems. Adding a dependency could result in the framework not functioning properly.

#### 4.1.3. Event Storage

It is responsible for storing and retrieving data from and for the event processing. The data can be stored locally or in a distributed manner. The size should be defined according to the domain or system need. However, some devices may not be able to store information, or the system may not be interested in keeping a record of its state, in which case this component can be disabled, making it optional.

#### 4.1.4. Event Output Channel

This component acts to provide events to the external environment. Along with the event input channel component, this component provides an extensible interface to support different communication protocols. It is possible to send the event to the next FaTEMa and the local F.T. from these interfaces.

### 4.2. FaTEMa Operation

**Figure 2:**
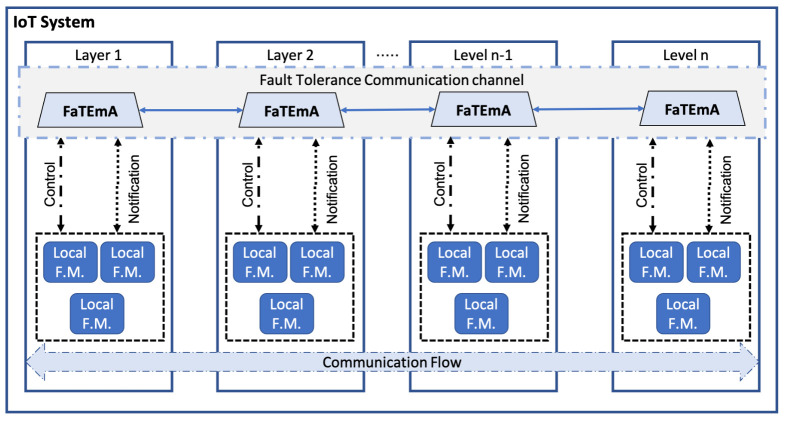
The multi-layer fault tolerance approach using FaTEMA.

The proposed framework creates a “virtual” communication channel across the different levels of abstraction, as shown in Figure 2. This channel facilitates information exchange through local fault tolerance mechanisms (Local F.M.) present in system levels. Each layer is subjected to specific types of uncertainty and different types of faults which have to be detected and recovered, ideally in a coordinated manner. Local fault tolerance mechanisms are responsible for handling faults in a specific layer or scenario without sharing a global system vision. Additionally, as IoT systems grow in size, complexity, speed, and heterogeneity, it becomes impractical to assume that a single fault tolerance mechanism can access an entire system’s information. Therefore, FaTEMa integrates system levels, enabling information exchange between fault tolerance approaches.

The framework’s assumption is to provide a decentralized mechanism to assist and support fault tolerance, particularly fault detection and recovery. Hence, there is no centralizing component that performs fault management. Each layer should be responsible for using the information obtained through FaTEMa and correlating them according to the context in which it is inserted. Thus, Local F.M.s must register in the framework and indicate the interest event types. Once an event is inside the communication channel, the interested Local F.M.s will receive and execute the appropriate actions to avoid the propagation of failures.

The introduction of mechanisms can increase a system’s complexity. The more significant the complexity is, the more susceptible to failure. This phenomenon in a system will lead to decreasing dependability. Additionally, if each tolerance mechanism provides a means to enable multi-layer information sharing, it will make the design and execution of such mechanisms even more complex. In this way, FaTEMa acts as an intermediary, reducing the general complexity of the communication process through the availability of the multi-layer communication channel. Therefore, fault tolerance mechanisms do not need to worry about this additional complexity by focusing on providing the best error detection and recovery strategy for their layer. Furthermore, the introduction of FaTEMa to a given system does not affect its current operation, since all communication is asynchronous and non-blocking. The framework supports two generic types of events: control and notification. Control event messages are related to fault-tolerance measures and treatments. Notification event messages carry information about the system’s current state, administrative information, or metrics regarding system utilization information.

The communication flow occurs in both directions. Higher-layer fault tolerance techniques can communicate with lower layers and vice versa. Additionally, FT techniques in the same layer can use the framework to collaborate in the measures against errors. However, to enable this intercommunication, there must be at least one FaTEMa instance at each layer. FaTEMa allows instance replication in order to assure availability and avoid single failure points. Each instance acts isolated. Nonetheless, it is possible to configure a distributed database to replicate the stored information between instances. These functionalities are available by default. Furthermore, all internal or external communication carried out by FaTEMa is executed asynchronously and non-blocking. Therefore, to send or receive FaTEMa events, each local FT must register in the framework’s input and output channels.

### 4.3. Running Example

**Figure 3:**
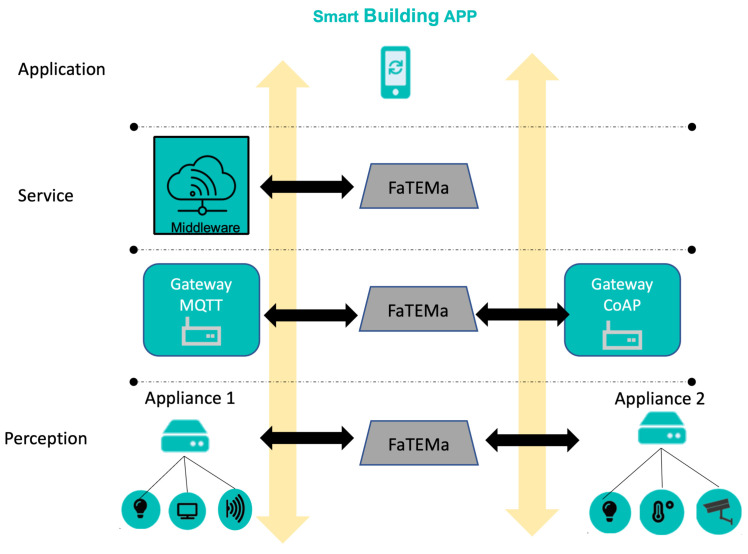
Example of a smart building scenario using FaTEMa.

Figure 3 shows how FaTEMa works in an illustrative IoT system for a smart building. The system architecture is organized into layers, in which the lowest layer has the physical devices (sensors and actuators) that interact with the environment. Devices are bound to appliances. In this example, we consider just two appliances connected to a set of devices, such as lamps, access points, temperature sensors, surveillance cameras, and screen monitors. The appliances are responsible for obtaining information from the sensors, monitoring their operation, and making the collected data available to the upper layers. The communication layer is responsible for centralizing the building information and sending it to the cloud middleware. In this example, the communication has two gateways, one for each communication protocol used by the appliances.

The service layer contains the middleware responsible for storing historical information, providing rapid computation, and combining information. In addition, middleware is the frontier to accessing the system from the outside world. Additionally, the middleware contains all device configurations and interactions. Finally, the application layer contains the client applications to monitor and interact with devices.

**Figure 4:**
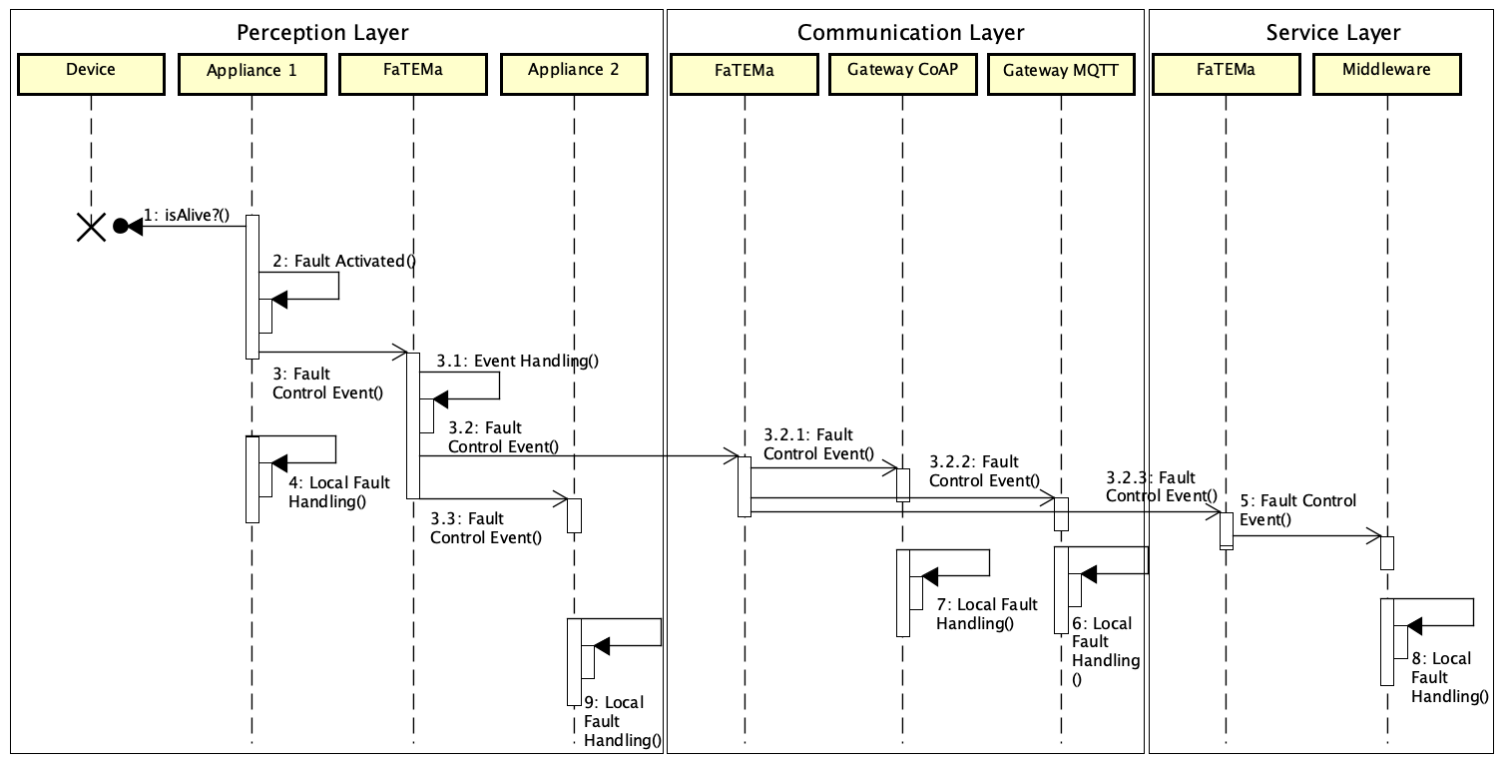
Communication event flow between layers in the smart building scenario.

Figure 4 shows the flow of messages related to error detection and its propagation through the use of FaTEMa. When the appliance identifies that a particular device has failed, it produces and sends a fault control event to the nearest FaTEMa. In parallel to the submission, the appliance is already applying fault tolerance mechanisms to prevent the error from propagating. Once the event is received in FaTEMa, it is processed, obtaining information regarding the origin and cause of the error. The event will then be stored for historical logging and error mapping. Then, each output channel identifies what is interested in the event and sends it. FaTEMas will always be interested, so all of the FaTEMa instances mapped in the configuration will receive the event. At the perception layer, Appliance 2 registers as interested in the control events. Therefore, every event received in the FaTEMa of this layer will be shared with it. Finally, the event is sent to the FaTEMa in the upper layer.

When the event arrives at the communication layer FaTEMa, it will be processed just as described in the previous layer. Once it is processed, the event is sent asynchronously to the gateways and to the FaTEMa of the next layer. Each gateway applies the necessary measures to avoid possible consequences regarding the error occurrence. The same behavior occurs in the next layer. The FaTEMa service layer receives and processes the event and makes it available to the fault tolerance mechanism present inside the middleware. Thus, an event is propagated across the layers, allowing this information sharing to reduce the propagation and occurrence of failures, making the system more reliable.

## 5. Experimental Evaluation

Aiming to evaluate the proposed framework, we established an empirical evaluation to assess the benefits of using FaTEMa on availability and reliability in IoT systems. For this, we used a smart home system comparing state-of-the-art fault tolerance mechanisms using or not FaTEMa. This system was designed considering several characteristics and requirements normally present in most IoT systems. This study aims to evaluate the potential of using FaTEMa to (a) improve error detection and service continuation capabilities, (b) enhance availability, and (c) increase reliability. To assess the benefits of introducing FaTEMa, we have collected a series of metrics in Table 2.

### 5.1. Experiment Design

**Figure 5:**
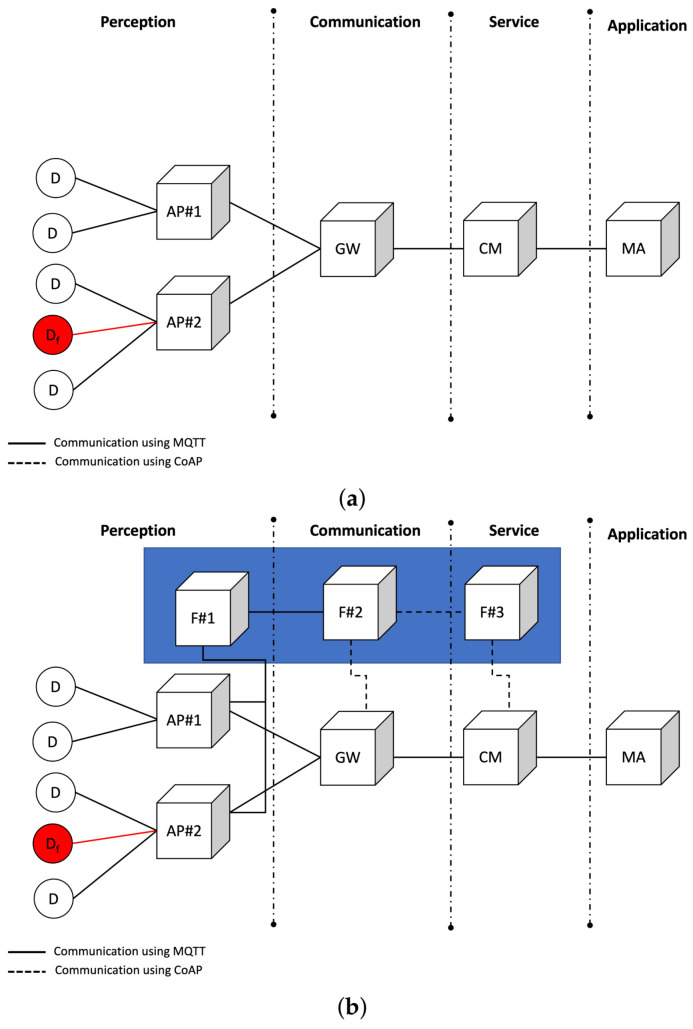
System architecture for experimental evaluation. (**a**) Experimenting system architecture without FaTEMA. (**b**) Experimenting system architecture with FaTEMA.

In order to carry out this evaluation, the simulated system was designed to involve monitoring devices in a home, such as security cameras, temperature, pressure, humidity sensors, and a variety of equipment (such as TVs, microwaves, and refrigerators). In particular, we performed a comparative evaluation with two different system instances, as shown in Figure 5.

The architecture is divided into four layers, as shown in Figure 5a. The perception layer is directly related to the device monitoring and actuation in the smart home. This layer contains a set of appliances connected to the monitored devices, making it possible to manage them. All information generated from or obtained by the house devices flows through a gateway that concentrates the information of a certain set of houses, connecting them to the upper layers. The service layer provides a cloud middleware that enables the treatment, processing, and storage of a high volume of information. Finally, a mobile application that interacts with the devices to manage them is available in the application layer. The executions that make use of FaTEMa have their architecture shown in Figure 5b.

The smart home contains several devices (D), sensing and acting in the environment to facilitate a user’s tasks. These devices are linked to an appliance (AP) responsible for monitoring and connection distribution. The appliances are responsible for having physical connections with devices, providing efficient communication with the rest of the system. The access to devices and appliances must happen through a gateway (GW) responsible for centralizing several instances of houses and transferring them to the upper levels. In addition, it receives requests directed to devices and identifies which paths the requests will follow. The cloud middleware (CM) has a high computational demand to process and store all the information transferred. Furthermore, it implements fault tolerance mechanisms in order to prevent system failures. The mobile application (MA) interacts with the CM, sending and receiving information to the devices. In this experiment, each system instance (devices, appliance, gateway, cloud middleware, and applications) ran independently on a different node.

The experiment was designed considering two scenarios using different fault tolerance mechanisms, as presented in Table 3. In S#1 the FT strategy operation consists of when the CM is informed that the requested resource is unavailable; then all new requests addressed to the faulty device are intercepted and returned, declaring its unavailability. Thus, the system is protected from causing new failures due to this unavailability. Simultaneously, the check mechanism using retry with exponential backoff is activated. Thus, the CM checks from time to time whether the resource resumed is in operation. Once it is identified that the resource is up and running, the checking process is stopped, and access to the resource will no longer be intercepted by the CM.

In S#2, a different check strategy was used in relation to S#1—active monitoring. Unlike S#1, where a failure is discovered only if the resource is requested, constant monitoring is executed to obtain information related to errors in all layers. The gossip-style error detection technique consists of monitoring each layer and the devices reporting whether they are active in a specific period. In addition, each layer checks at periodic intervals whether any devices on the list have not sent the heartbeat signal. If the exceeded defined time limit is reached, the device is considered faulty, and this information is propagated across the layers. When the CM receives a new incoming request for the unavailable device, it then intercepts and returns, informing its unavailability, avoiding new failures. When the resource is back online, the interception ceases.

To compare the benefits introduced by FaTEMa in an IoT system, each scenario was executed using FaTEMa and without using FaTEMa. This introduction of FaTEMa occurs when an error is detected. AP#2 sends a control event informing the CM that a specific device has failed to the nearest FaTEMa at the layer. Once the event is received, it is propagated through the layers until it reaches the CM. Consequently, when the CM identifies the error occurrence, it activates the request interception mechanism of new requests to the faulty device preventing new failures. Furthermore, as soon as AP#2 detects that the device has resumed its operation, it propagates the event using FaTEMa, and as soon as the CM detects that the device has resumed its operation, the following requests will not be intercepted.

### 5.2. Execution

In order to evaluate the benefits of using FaTEMa, this experiment injected two omission faults in the system at specific intervals. The faults occurred in a device (temperature sensor) connected to AP#2, occurring according to the following pair (start, duration): FA1—fault activation 1 (5, 10); FA2—fault activation 2 (15, 25). The start time means the instant of the fault activation, initiating the omission errors. The duration is the amount of time the error occurrence lasts (both expressed in seconds). The values of request timeout and the maximum throughput were 200 ms and 20 req/s, respectively. In the beginning of the test, a setup phase was executed to minimize the resource initialization effects. This procedure took approximately 5 s, and only after this, the MA started to send requests for 30 s. Each scenario was executed 20 times, and, in each one, all requests were randomly generated. Thus, measures obtained and analyzed were the averages of these executions.

For the physical nodes, we used Raspberry Pi 3 Model B, interconnected in a network of 100 Mbps. Additionally, the systems were written in Java, and the communication protocols adopted were CoAP (Constrained Application Protocol) and MQTT (Message Queuing Telemetry Transport). The protocol specification used by each system entity is depicted in Figure 5. The software implementation was based on the Spring Boot framework, while the communication protocol implementation used for CoAP was the open-source library Californium (Californium—https://www.eclipse.org/californium/, accessed on 10 October 2021) and for MQTT was the open-source library ActiveMQ (ActiveMQ—https://activemq.apache.org/mqtt, accessed on 10 October 2021). In terms of behavior, the mobile application instances requested the CM, which also requested cascading GW and APs. The fault tolerance strategy was only included in the CM, which is the system entry point, which has greater computational power with fewer energy restrictions and communication availability.

### 5.3. Results

To assess the benefits of using FaTEMa to improve error detection and service continuation, we collected the average error detection and resolution times for each scenario, as presented in Table 4 and Table 5. In each table, for each scenario, executions with FaTEMa (WF) and without FaTEMa (WOF) are compared. Error detection time in the scenario with FaTEMa demonstrated a decrease in the interval between the error occurrence and its detection, regardless of the scenario. The difference in S#1 was 10.47% and in S#2 was 45.71%. The perceived difference in S#1, although not significant when compared to S#2, reflects a notable improvement in error detection time. The experiment considered a limited number of devices, making it more probable to request information from a faulty device, which advocates decreasing error detection time in runs without FaTEMa. However, when compared to a real scenario where there are hundreds of thousands of devices, the intervals and the frequency of requests can be much higher and more numerous, respectively, which would lead to a worsening in the error detection time.

In S#2, the error detection strategy used fault information propagation between layers. This propagation directly competed with the information processing that exists in the layers. Thus, it could directly impact the propagation time of information about the error, causing delays in the detection time. This fact may explain the perceived difference in error detection time in the executions that used FaTEMa.

Regarding the error resolution time, in S#1, a difference of 8.7x was noticed by the executions without FaTEMa. This difference can be explained by the large processing time perceived in FA2. The error time in FA2 is double in relation to FA1, and the fault tolerance strategy uses the retry check with exponential backoff, which explains the exponential increase in the recovery time. Thus, we realized that the longer the error time, the longer the system’s recovery time. In S#2, the decrease in error resolution time was 22.49% in runs with FaTEMa. Despite using the same propagation strategy, the error detection time was considerably higher than the error resolution time in the executions without FaTEMa. One factor contributing to the reduction in the resolution time is the decrease in the throughput of requests within the system, because with the fault tolerance strategy activated, requests addressed to the faulty device were intercepted and returned. Thus, the competition between fault information propagation can be impacted, depending on the current processing of the system. This behavior was not noticed in the executions with FaTEMa because the difference between the recovery times was not significant, and there was no direct competition with the system’s functioning.

To evaluate the availability benefits of using FaTEMa, we measured the system’s availability time. This metric was obtained through the relation between (TA-TD-TR)/TE, where TA is the hypothetical time in which the system correctly answers the requests, and is the experiment time. Independently of the scenarios, the faulty device availability (Ad) was fixed based on the experimental configuration (TA = 30 - 5 - 10, TE = 30, and consequently Ad = 50%). TD is the average error detection time (see Table 4) and TR is the average error resolution time (see Table 5. The measurements and intermediary metrics are presented in Table 6.

The results obtained indicate that FaTEMa executions achieved higher availability time compared to not using it. This result can be explained by the fact that decreases in error detection and resolution time will consequently increase system availability time. In S#1, there was a significant difference in the availability time, since the retry strategy used required a long time to identify the error recovery application, which had a high impact on system availability. In S#2, there was no significant difference in the availability time between executions. However, the FaTEMa executions improved the availability time due to reducing error detection and resolution time.

In order to assess the benefits of introducing FaTEMa, we collected responses to requests received in the system. The obtained data are summarized in Table 7. The results revealed that despite the introduction of FaTEMa, no significant impact on the total number of requests was perceived. As illustrated in S#2, the number of requests in the executions with FaTEMA was higher than than the number of those without FaTEMa. Furthermore, in the comparison between scenarios, it is possible to notice that the use of FaTEMa reduced the numbers of errors and failures produced and increased the number of errors detected.

To perform the system reliability assessment, we derived some metrics from the primary data. The failure rate metric [66] was established by the total of failures/Te, indicating the failure rate over a given time. Executions using FaTEMa achieved a considerably lower failure rate in both scenarios. Another way to analyze reliability is by metrics that relate faults, errors, and failures [67]. Thus, we used the following set of metrics: percentage of failures (total of failures/total of injected faults)—identifies how many failures were produced from the total injected faults; error detection coverage (total of detected errors/total of errors)—indicates how many of the activated errors were detected (that is, the greater the coverage, the fewer failures should be produced); percentage of error activation (total errors/total of injected faults)—indicates how effective the fault tolerance strategy can be at preventing errors from being produced.

In both scenarios, the use of FaTEMa demonstrated increases in system reliability metrics. Furthermore, the improvement in the error detection coverage metric, noticed in the executions with FaTEMa, increased the reliability due to the reduction in the occurrence of failures.

### 5.4. Discussion

The experimental results indicated that FaTEMa is able to improve the error detection and resolution process, reducing the time required for detection and return to the system’s regular operation. Consequently, an improvement in the system’s availability time could also be observed. Moreover, the results showed that it effectively improves reliability attributes, decreases failures, improves error detection coverage, and maximizes the rate of successful requests.

**Figure 6:**
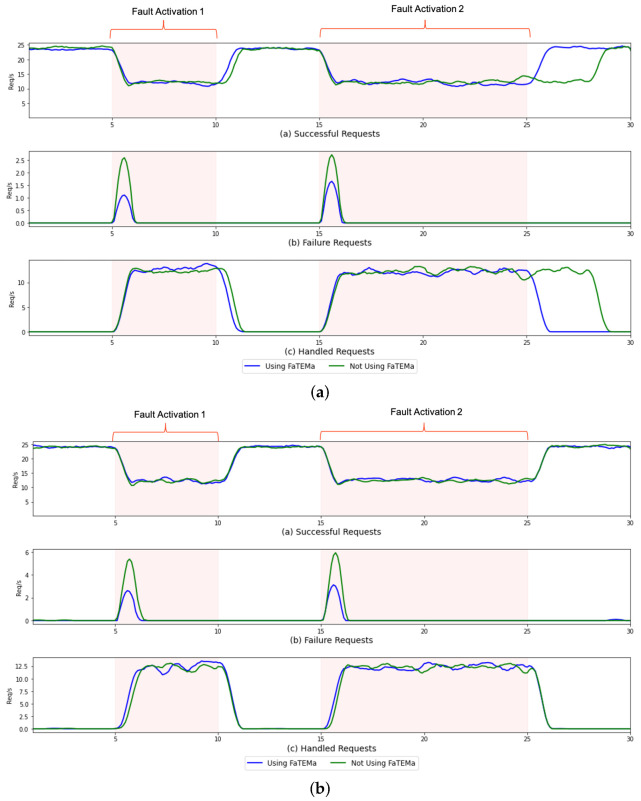
A comparison of the responses over time for each experimental scenario. (**a**) S#1—responses over time. (**b**) S#2—responses over time.

Figure 6 presents a comparison over time of the system responses in each execution for each scenario that supports this finding. In both scenarios, it is possible to notice that the use of FaTEMa decreased the number of failures and activated the fault tolerance strategy more quickly, demonstrating the importance of obtaining an efficient error detection mechanism. In Figure 6a, we can see the impact caused by the fault tolerance strategy based on bounded retries in the error resolution and system service continuation. FaTEMa made it possible to minimize this impact by reducing the time taken for the system to resume regular operation. S#2 scenario, as shown in Figure 6b, demonstrated that using FaTEMa, even with a proactive detection and recovery strategy, improved the failure detection process. This improvement can be explained by the independence of the framework in relation to the system’s functioning. The use of FaTEMa allows the error detection and recovery process to occur independently of the technique or algorithm used in the system layers.

A highlighted aspect supported by the results is that the introduction of FaTEMa made it possible to enable proactive communication in error detection and error detection resolution stages without increasing the system’s complexity. As a result, the reactive communication model for error detection and resolution could remain active and be combined with a proactive supported by FaTEMA. In addition, enabling a multi-layer communication channel made it possible to support the exchange and combination of fault tolerance information between layers. Consequently, it was able to replicate this information, achieving redundancy. This replication and redundancy benefit the system, allowing distributed fault tolerance decision-making. In this way, it is possible to combine strategies so that the decision regarding fault management considers the needs and requirements of other layers. A key point identified in the FaTEMa approach is that, in order for a failure to be tolerated, the first step is to identify it. Thus, the more layers that obtain this information, the more effective its treatment will be. Therefore, using an exclusive, asynchronous, and event-oriented communication channel proved to be an effective strategy for fault detection and recovery in IoT systems.

In this experiment, MQTT and CoAP communication protocols were used, as shown in Figure 5. Thus, it was possible to demonstrate that FaTEMa supports adaptation to new technologies and communication protocols, supporting the heterogeneity in IoT systems. Furthermore, introducing a new fault-tolerance approach will not increase the system’s complexity. Therefore, the introduction of FaTEMa did not show impacts on the overall functioning of the system, as can be seen in Table 7. The total number of requests in the executions have very similar values, and its variation can be explained by the randomness used in the experiment.

**Figure 7:**
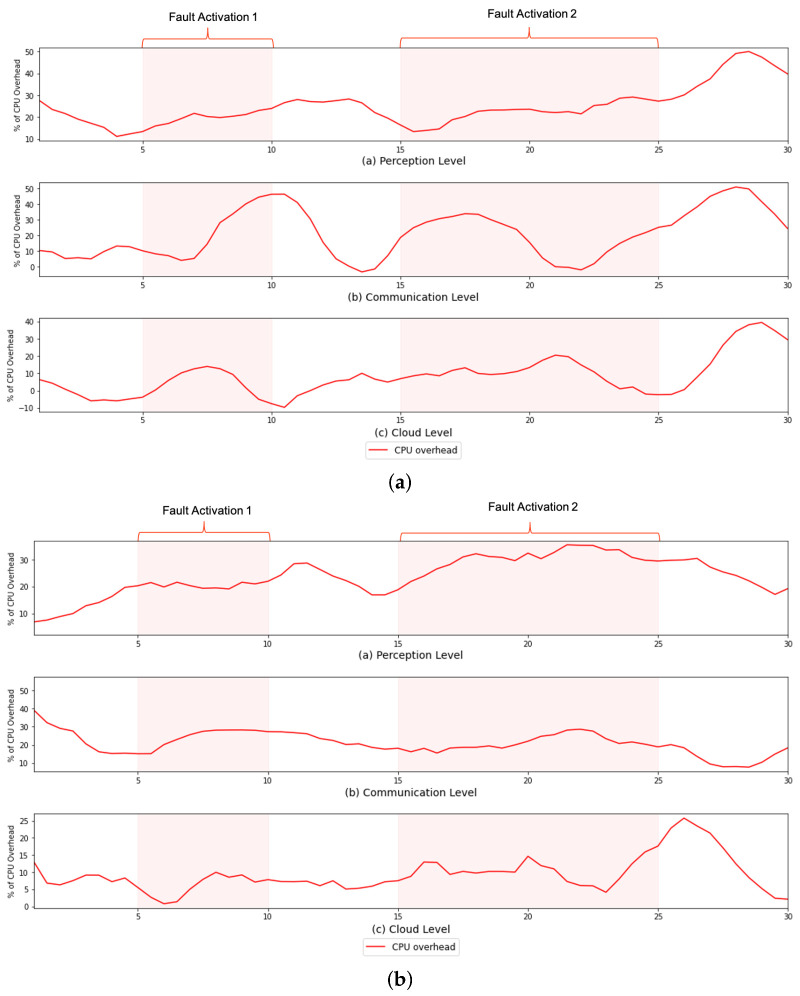
A comparison of the CPU overhead over time in each experimental scenario. (**a**) S#1—CPU overhead over time. (**b**) S#2—CPU overhead over time.

**Figure 8:**
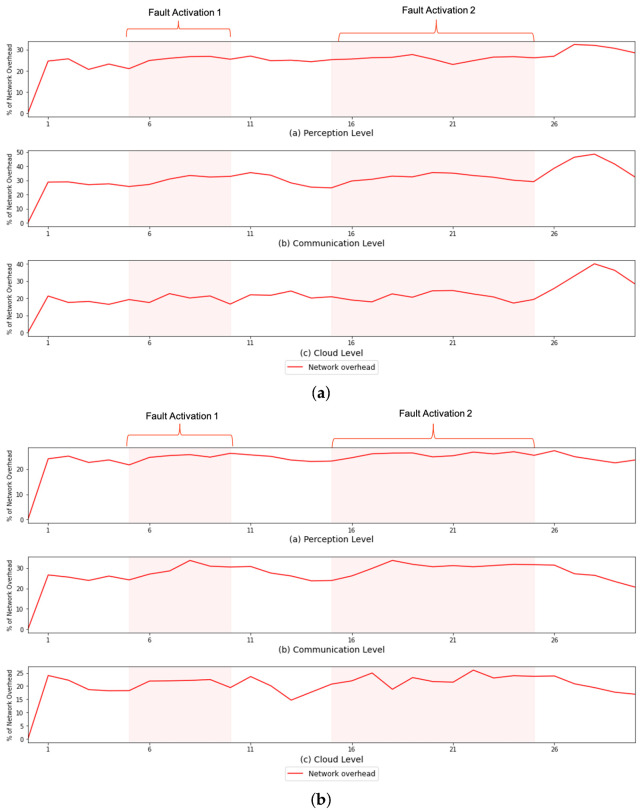
A comparison of the CPU overhead over time in each experimental scenario. (**a**) S#1—network overhead over time. (**b**) S#2—network overhead over time.

However, despite not impacting the system’s overall functioning, the use of FaTEMa increased the need for processing resources and bandwidth. Figure 7 and Figure 8 illustrate the CPU overhead and network bandwidth, respectively, over time caused by the introduction of FaTEMa. The impact on CPU usage is most noticeable for the communication layer and perception layer. For both scenarios, it is possible to notice this behavior. However, a lower impact was perceived at the cloud layer. In some periods, there was no overhead caused by the use of the framework. This CPU overhead can be explained by the fact that the cloud layer performed the most processing, which did not increase the impact caused by FaTEMa. Thus, there are indications that the introduction of FaTEMa caused in scenarios where there was little processing, a higher CPU overhead. Regarding bandwidth use, there was a pattern of an around 20–30% increase regardless of the scenario or layer. In this way, there is an indication that an increase of bandwidth overhead will depend directly on the volume of information exchange between the layers.

### 5.5. Experimental Remarks

Although the results presented in this experiment demonstrate the feasibility of using FaTEMA as a dedicated channel for transmitting events related to fault tolerance, only two scenarios were considered. However, the scenarios used considered the main characteristics present in real-world IoT systems to guarantee the study’s validity. Furthermore, fault tolerance mechanisms used and popular in distributed systems were considered. Besides, it was evidenced that the exchange of collaborative messages about tolerance between the layers of the IoT system can assist and improve error detection and error recovery. Consequently, FaTEMA improves availability and reliability attributes. The creation of the multi-layer communication channel enables information redundancy, promoting end-to-end system fault tolerance. It also supports distributed decisions, facilitating the management of specific requirements present in the layers. Therefore, we believe that the entities closest to the errors are best to identify error occurrence and propagate the information across layers in a simplified way. Therefore, this approach leads to FT information exchange to produce fault tolerance strategies that adapt to the contexts where they are inserted and collaboratively interact to tolerate a greater number of faults producing more reliable IoT systems.

The introduction of FaTEMa presented significant advances in relation to existing proposals in the literature. As presented in Section 2, the proposal by Woo et al. [25] focused only on the healthcare area; and unlike the aspects demonstrated in this experiment, it does not have the means to attain heterogeneity aspects present in IoT solutions. In addition, it focused on recovering failures through information propagation to enable node recovery. However, each node only knows the information from nodes of the same layer or higher. In our proposal, as demonstrated in the experiment, the information regarding fault detection and error recovery is propagated to the entire system, enabling a distributed decision-making process.

The proposal presented by Su et al. [57] did not demonstrate having ways of dealing with heterogeneity or enabling interoperability between different protocols or technologies. The FaTEMa usage allowed it to meet different technologies and protocols and enable information sharing between heterogeneous layers, providing interoperability. Furthermore, the results shown by the authors do not show what improvements were achieved in terms of availability and reliability. FaTEMa indicates that its use increased the availability and reliability of the evaluated system. Furthermore, FaTEMa acts independently of the system’s functioning. That is, it does not introduce any additional complexity. In Su et al.’s proposal, no evaluation was conducted to assess the fault tolerance mechanism’s impact on the system regarding complexity and performance.

## 6. Conclusions

IoT systems are highly distributed and heterogeneous. These characteristics and the interdependence between system layers make using a unified fault tolerance strategy a challenge. Thus, this paper proposes the FaTEMa framework to enable the propagation of fault tolerance information events across layers of an IoT system. It acts as a mediator, enabling communication to facilitate collaboration between layers and its local fault tolerance, aiming to improve error detection and continued service. Thus, the proposed framework focuses on assisting error detection and fault treatment and continued service, especially resuming a system to normal service. Moreover, this framework provides a multi-layer approach enabling an end-to-end communication channel that facilitates FT event exchange, providing information redundancy to minimize fault occurrence and propagation effects. In addition, the framework facilitates interoperability between diverse fault tolerance mechanisms by defining a communication standard.

The empirical evaluation was accomplished by experimenting with two scenarios using a well-known fault tolerance approach. Every scenario was compared with and without FaTEMa. Overall, the results show that the use of FaTEMA improved error detection and error resolution. Additionally, a benefit was observed due to the framework’s introduction: improving the system availability. The improvement in the error detection step was demonstrated to cause a significant impact on improving system reliability and availability. Once an error is detected, measures to tolerate and recover faults are activated. Equally important is the effectiveness of resuming regular system operation once a fault is recovered. The use of FaTEMa demonstrated a high capacity to propagate the recovery event between layers, enabling more efficient resumption of the system’s normal functioning, leading to improved availability. In addition, our experiments showed that the FaTEMa improved the fault tolerance’s effectiveness on failure rate, error activation, and error detection.

Such results suggest that our multi-layer fault tolerance approach using FaTEMa could improve the overall system reliability and availability due to the following reasons: (a) a proactive and unified communication schema allowing information exchange regarding fault tolerance events, assisting in error detection and service continuation; and (b) the possibility of collaboration between different layers to obtain the best fault tolerance strategy according to the context, providing end-to-end fault tolerance in the IoT system.

As future work, from our work emerges the need for new fault tolerance mechanisms using the combination of information between layers to enable a better understanding of faults. Furthermore, given the collaboration between systems, it is essential to evaluate and enable sharing of fault tolerance information inter-systems, reducing the effects caused by the propagation of failures.

## Figures and Tables

**Table 1:** Challenge addressed by the related work.

Related Work	CH1	CH2	CH3	CH4
Fortino et al. [54]	No	Yes	No	No
Abreu et al. [55]	No	Yes	Yes	No
Woo et al. [25]	No	No	No	Yes
Guimaraes et al. [33]	No	No	No	Yes
Li et al. [18]	No	No	Yes	Yes
Belckacem et al. [56]	No	No	Yes	Yes
Su et al. [57]	No	No	Yes	Yes
Our approach	Yes	Yes	Yes	Yes

**Table 2:** Measures used to assess the benefit of using FaTEMa.

Benefit	Metric	Description
Error detection	Error Detection Time	The time to detect an error occurrence
Service Continuation	Error Resolution Time	The time from the error recovery application to its effective resolution.
Availability	System Availability Time	The time in which the system produces correct responses to requests made.
Reability	Successful requests	The number of successful requests.
	Activated errors	The number of faults that were activated.
	Failures	The number of errors that were perceived by the applications, that is, they surpassed the system’s borders.
	Detected Errors	The number of enabled errors that were detected and did not produce failures.

**Table 3:** Experimental scenarios and fault tolerance mechanisms.

Scenario	Fault Tolerance Mechanism	Executions
S#1	Bounded Retries [64]	Without FaTEMa
		With FaTEMa
S#2	Gossip-Style Error Detection [65]	Without FaTEMa
		With FaTEMa

**Table 4:** Error detection time measurements.

Scenarios		FA1 (ms)	FA2 (ms)	AVG (ms)
S#1	WF	172.5	187.55	180.03
	WOF	198	199.75	198.88
S#2	WF	244.2	287.75	265.98
	WOF	396.55	378.6	387.58

**Table 5:** Error resolution time measurements.

Scenarios		FA1 (ms)	FA2 (ms)	AVG (ms)
S#1	WF	212.35	195.85	204.10
	WOF	486.7	3090.75	1788.73
S#2	WF	205.75	215.65	210.70
	WOF	251.2	265	258.10

**Table 6:** System availability measurements.

Scenarios				
	**S#1**		**S#2**	
**Metric**	**WF**	**WOF**	**WF**	**WOF**
TE (s)	30.00	30.00	30.00	30.00
TA (s)	15.00	15.00	15.00	15.00
Ad	50.00%	50.00%	50.00%	50.00%
TD (s)	0.36	0.40	0.53	0.78
TR (s)	0.41	3.58	0.42	0.52
As	47.44%	36.75%	46.82%	45.70%

**Table 7:** Reliability measurements.

Metric (Avg)	S#1		S#2	
	WF	WOF	WF	WOF
Total	1166.6	1179.25	1191.05	1181.1
Sucessful	859.1	808.65	879.4	872.85
Tolerated	302.3	363.25	302.9	295.35
Injected faults	296.35	296.7	297.1	296.1
Error	5.2	7.35	8.75	12.9
Failures	2.85	5.55	6.1	12.55
Detected	2.35	1.8	2.65	0.35
Failure Rate	0.10	0.19	0.20	0.42
Percentage of Failures	0.97%	1.88%	2.07%	4.24%
Error Detection Coverage	45.19%	24.49%	30.29%	2.71%
Percentage of Activated Errors	1.75%	2.48%	2.95%	4.36%

## Data Availability

The data used to support the findings of this study are included in the article. More detailed resources were also publicly published, including source code and simulation results. These are available in the FaTEMa framework repository on Github (FaTEMa frameowork—https://github.com/mariovmelo/fatema-framework, accessed on 10 October 2021).
